# Diagnostic accuracy of diffusion weighted MRI in differentiating malignant from benign liver lesions taking histopathology as gold standard

**DOI:** 10.12669/pjms.40.4.7738

**Published:** 2024

**Authors:** Jia, Sumera Tabassum, Shahbaz Haider, Haania

**Affiliations:** 1 Jia Resident, Department of Radiology JPMC, Karachi; 2 Sumera Tabassum Associate Professor, Department of Radiology JPMC Karachi; 3 Shahbaz Haider Professor, Medical Unit-I JPMC Karachi; 4 Haania Research Fellow, Clinical Attachment, JPMC Karachi

**Keywords:** Focal liver lesion, Magnetic Resonance Imaging (MRI), Diffusion Weighted Imaging (DWI), Benign, Malignant, Histopathology

## Abstract

**Objective:**

To determine the diagnostic accuracy of Diffusion Weighted MRI in differentiating malignant from benign liver lesions taking histopathology as gold standard.

**Methods:**

This Cross-sectional study was conducted at Departments of Radiology and Medicine, JPMC, Karachi from February 23, 2019 till September 25, 2019. Data was prospectively collected from patients after taking consent. One hundred twenty five patients presenting with hepatic mass who met the inclusion criteria were included. Quantitative data was presented as simple descriptive statistics giving mean and standard deviation and qualitative variables as frequency and percentages. Sensitivity, specificity, positive and negative predictive values and diagnostic accuracy were calculated. P-value of ≤0.05 was considered as significant.

**Results:**

Mean age in our study was 59.75±8.57 years. Total 71 (56.8%) were male and 54 (43.2%) were female. Out of 125 patients, sensitivity, specificity, positive predictive value, negative predictive value and diagnostic accuracy of DW MRI for the diagnosis of malignant focal liver lesion by taking histopathology as gold standard was found to be 92.3%, 93.6%, 96%, 88% and 92.8% respectively.

**Conclusion:**

DW MRI scan has high diagnostic accuracy and being accurate in making a diagnosis and differentiation of benign from malignant focal liver lesion would decrease need of invasive modality of histopathology.

## INTRODUCTION

Focal liver lesions are more frequently picked and reported as compared to the past due to the increasing availability and use of imaging modalities like ultrasonography (US), magnetic resonance imaging (MRI) and computed tomography (CT). As technical standards of ultrasound equipment are improving and along with it, number of abdominal ultrasound imaging is increasing, the number of incidentally reported focal liver lesions, named ‘‘incidentalomas,’’ are also increasing remarkably.^1^ Focal liver lesions could be classified from benign with or without indication of any treatment and malignant lesions, thus falling into three clinical categories.^2^,^3^

Treating physicians are now having frequent cases where they have to decide which investigations are to be advised to conclude that under focus focal liver lesion is benign or malignant keeping cost ,availability and invasiveness of investigation in mind.^4^-^6^ Conventionally available “Histopathology of hepatic lesions is gold standard for differentiating benign from malignant lesions but as it is invasive”, different imaging modalities are being studied to decrease the need of Biopsies and surgeries.^7^ Magnetic resonance imaging with gadolinium contrast is mostly recommended as it has high resolution for soft tissues . Furthermore, it has the ability to characterize soft tissue lesion on different data acquired, such as T1, T2, weighted and post-gadolinium images taken early and late”.^8^,^9^

But recently in patients having some degree of renal insufficiency were reported to develop “nephrogenic systemic fibrosis (NSF)” due to administration of intravenous gadolinium contrast. This observation led to search of “novel MRI techniques” which would not need intravenous gadolinium.^10^

Diffusion-weighted imaging (DWI) is such a novel technique. It is “non-invasive and rapidly acquired technique and there is no need of intravenous gadolinium contrast. DWI works in biological tissues by measuring^10^,^11^ ‘random molecular motion’ which is thermally induced”.^10^-^12^ “The DWI sequences have been suggested as diagnostic tool which may be useful in diagnosis and differentiation of benign and malignant hepatic +lesions “.^13^,^14^

***Objective*** was “to estimate the diagnostic accuracy of Diffusion Weighted MRI in discriminating malignant from benign focal liver lesions taking histopathology as gold standard” in patients presenting at teaching Hospital in Karachi and “to compare local results with international studies and add to the international literature by our locally produced data in metropolitan city, Karachi, Pakistan”

### Operational definition:

### ADC values

“Apparent diffusion coefficient” abbreviated as ADC, is a value of “degree of diffusion of H_2_O molecules” in focused tissue, and is usually determined using MRI with DWI. The ADC value is determined and provided by MRI software after putting cursor on smallest Region of Interest (ROI) in needed area. Unit of ADC value is written as mm^2^/s (as an example value may be 2.0 to 2.1 x 10^-3^mm^2^/s). These values with certain cut off points are used for determination of benign and malignant character of focal lesions as below.

### Malignant focal liver lesions on diffusion weighted MRI

“Focal liver lesions measuring more than 1cm in diameter having variable signal intensity on T1 and T2 weighted images and commonly hypointense on DW MRI with ADC values of less than 1.5x10^-3^mm^2^/cm were labeled as Malignant Liver Lesions”

### Benign focal liver lesions on diffusion weighted MRI

“Focal liver lesions measuring more than 1cm in diameter having variable signal intensity on T1 and T2 weighted images and commonly hyperintense on DW MRI with ADC values of more than 1.5x10^-3^mm^2^/cm were labeled as Benign Liver Lesions.

## METHODS

This “cross-sectional study was conducted by the Department of Radiology and Medicine, Jinnah Postgraduate Medical Center, Karachi from 23-02-19 to 25-09-19”. The sample size was “calculated by taking sensitivity 92.8%, specificity 91%, disease prevalence 42.7%, desired precision 0.07 and 95% confidence interval”. These values were entered in web sample calculator, available at web site: https://wnarifin.github.io/ssc/sssnsp.html “The calculated sample size tuned to be 125patients.Non-probability consecutive sampling technique was used.

### Ethical Approval

The study was “approved by the Ethical Review Board of Jinnah Postgraduate Medical Center (Duplicate letter no: F2-81Genrl/262/JPMC Dated: December 31, 2022)”.

Patients of either gender, with age from 30-70 years having focal liver lesion as detected on ultrasound examination and referred by physicians were included in the study. Non-consenting patients, Pregnant patients, Patients with metallic implants and cardiac pacemakers, as these foreign bodies are contraindications for MRI, and Patients having simple liver cysts on ultrasound appearing as “well defined anechoic lesions with posterior acoustic shadowing” were excluded. Patients whose “biopsy could not be done or report could not be collected were also excluded from the study”. Patient demographics and clinical history was taken by the investigators and recorded on Proforma designed for the study.

“Diffusion Weighted MRI for differentiation of liver lesions was done on Phillips 1.5 Tesla MRI Scanner, using 8-channel high resolution head coil. Axial T1, T2, Sagittal T2 and coronal FLAIR images with slice thickness of 5mm, was retrieved. In the transverse plane a single-shot spin-echo echo- planar DW imaging sequence was obtained with three orthogonal diffusion gradients”. Scan was assessed by two senior consultant radiologists on console with post fellowship experience of greater than five years and assisted by other researchers.

All cases with Lesions diagnosed as malignant or benign on DW-MRI findings were advised for biopsy and their histopathological reports were collected. Clinical history, Ultrasound findings, findings of the MRI scan and final diagnosis of histopathological report of each patient was entered in the proforma.

### Data analysis procedure

Data was entered in specifically made database in SPSS 22 for windows. Mean and standard deviation was computed for quantitative variables like age, size of focal liver lesions in centimeters. Frequency and percentage were calculated for malignant focal liver lesion on DW MRI and on histopathology” and similar calculations were done for benign lesions. The diagnostic accuracy of DWI (in terms of ADC values) for characterization of suspected liver lesions in differentiating malignant from benign lesions was determined along with positive predictive value (PPV), negative predictive value (NPV), sensitivity and specificity, taking histopathology as gold standard.

## RESULTS

A total of 125 patients visiting Departments of Radiology and\or Medicine, JPMC, Karachi after satisfying the inclusion and exclusion criteria were included in this study. Age range among these 125 patients as seen was from 34 years minimum to 69 years maximum. Among these 125 patients, male were 71 (56.8%) while 54 (43.2%) were female. Mean age in our study was 59.75 years with the standard deviation (SD) of ±8.57. Out of 125 patients, 32 (25.6%) patients were in age group of 30-50 years while 93 (74.4%) were in older age group of 51-70 years. DW MRI showed that out of 125 patients, 75 (60%) focal liver lesion were malignant and 50 (40%) were benign in nature. While on histopathology out of 125 patients, 78 (62.4%) focal liver lesion were malignant and 47 (37.6%) were benign in nature.

Frequency distribution of cirrhosis status showed that out of 125 patients, 25 (20%) had cirrhosis while 100 (80%) patients did not have cirrhosis. Out of 125 patients, sensitivity, specificity, PPV, NPV and diagnostic accuracy of DW MRI for the diagnosis of malignant focal liver lesion by taking histopathology as gold standard was found to be 92.3%, 93.6%, 96%, 88% and 92.8% respectively.

On stratification for age with respect to sensitivity, specificity, PPV, NPV and diagnostic accuracy of DW MRI for the diagnosis of malignant focal liver lesion by taking histopathology as gold standard in age group 30-50 years was found to be 92.8%, 75%, 96.2%, 60% and 90.6% respectively. Moreover, in age group 51-70 years these were found to be 92%, 95.3%, 95.8%, 91.1% and 93.5% respectively.

Stratification for gender with respect to sensitivity, specificity, PPV, NPV and diagnostic accuracy of DW MRI for the diagnosis of malignant focal liver lesion in male group were found to be 89.3%, 92%, 95.4%, 82.1% and 90.2% respectively. While, in female group these were found to be 87.5%, 98.5%, 87.5%, 98.5% and 97.4% respectively. Parameters after stratification for *status of cirrhosis* of malignant focal liver lesion on DW MRI are presented in Table-I. Parameters after stratification for size of malignant focal liver lesion on DW MRI are presented in Table-II.

**Table-I:** Diagnostic accuracy of diffuse weighted MRI for the diagnosis of malignant focal liver lesion by taking histopathology as gold standard according to cirrhosis status n=125.

Cirrhosis Status	DW MRI	Histopathology		Total	

		Yes	No		
Yes	Yes	06 (TP)	02(FP)	08	SEN 75%
	No	02(FN)	14(TN)	16	SPE 87.5%
	Total	08 16		24	PPV 75%
					NPV 87.5%
					DA 80%
No	Yes	66(TP)	01(FP)	67	SEN 94.2%
	No	04(FN)	29(TN)	33	SPE 96.6%
	Total	70 30		100	PPV 98.5%
					NPV 87.8%
					DA 95%

**Table-II:** Diagnostic accuracy of diffuse weighted MRI for the diagnosis of malignant focal liver lesion by taking histopathology as gold standard according to diameter of malignant focal liver lesion on DW MRI n=125.

Size of malignant focal liver lesion on DW MRI	DW MRI	Histopathology		Total	

		Yes	No		
<3 cm	Yes	42(TP)	02(FP)	44	SEN 6.3%
	No	05(FN)	11(TN)	16	SPE
	Total	47 13		60	84.6%
					PPV 5.4%
					NPV 8.7%
					DA 88.3%
>3 cm	Yes	30(TP)	01(FP)	31	SEN 6.7%
	No	01(FN)	33(TN)	34	SPE 97%
	Total	31 34		65	PPV 6.7%
					NPV 97%
					DA 95.3%

## DISCUSSION

MRI with gadolinium contrast is considered the most accurate imaging technology, but as mentioned in introduction, recently after being given intravenous gadolinium contrast in patients having some degree of renal insufficiency were reported to develop “nephrogenic systemic fibrosis (NSF)”.^10^ This observation led to search of “novel MRI techniques” which would not need intravenous gadolinium. DWI is such a novel technique. It is “non-invasive and rapidly acquired technique and there is no need of intravenous gadolinium. “The DWI sequences have been suggested as diagnostic tool which may be useful in diagnosis and differentiation of benign and malignant hepatic lesions”.^13^,^14^ DWI MRI has also been employed with encouraging results in discrimination of different meningiomas and diagnosis of encephalitis.^15^,^16^ This DWI for liver lesions and masses is being studied and discussed in this article.

Our study showed that mean age was 59.75±8.57 years. 71 (56.8%) were male and 54 (43.2%) were female. Taking ADC values of less than 1.5x10^-3^mm^2^/cm along with other points given above as diagnostic clues for malignant liver disease, and taking histopathology as gold standard, out of 125 patients, sensitivity and specificity for the diagnosis of malignant liver lesion were found to be 92.3%, 93.6%. Taking same criteria PPV and NPV of DW MRI were found to be 96%, and 88% respectively. Diagnostic accuracy of DWI for the diagnosis of malignant liver lesion was found to be 92.8%.

Stratification for cirrhosis status with respect to diagnostic accuracy, sensitivity, specificity, PPV and NPV of DW MRI (Table-I) show that these values are lower in patients with cirrhosis. Similarly, stratification for size of malignant focal liver lesion showed lower values in size group < 3cm, a factor to be kept in mind (Table-II). Latife et al study^2^ “included 60 liver lesions which were scanned using 1.5 T MRI. Mean of ADC values of both groups of benign and malignant lesions (on histopathological based division) were compared. Reference standards were obtained for both groups on histopathologic findings. Different close values were also analyzed. When ADC cut off value of 1.0 × 10^-3^ mm^2^/s was analyzed for diagnosis of malignancy; sensitivity, specificity and accuracy came out to be 90.3%, 78.57% and 86.7% respectively”. The best correlating result with histopathology was gained when cut off value of ADC was settled at 1.5 × 10^-3^ mm^2^/s as results got were 90.3% sensitivity, 92.86% specificity, 91.1% accuracy, 96.6% PPV and 81.3% NPV. It also concluded that DWI and ADC cut off value determination is a prospective tool in differentiating malignant from benign liver lesions.^2^

Another prospective study by Hasan NM et al.^17^ “included 40 consecutive patients. These patients were having 64 focal liver lesions. They were investigated by having MRI of the liver. All patients had one or more hepatic lesions with diameter of more than 1 cm. In this study qualitative assessment by DWI and Quantitative assessment employing ADC map were also compared. Quantitative method using ADC values proved more precise (87.5%) than qualitative method for reliably labelling different focal liver lesions (FLLs) as benign or malignant.

Malignant lesions had Mean ADC values of 0.94 + 0.32 × 10^-3^ mm^2^/s, while benign lesions had values as 2.64 ± 0.46 × 10^-3^ mm^2^/s. Benign lesions may be seen to have clearly higher values thus helping in differentiation. Analyses in this study by Hasan NM had shown that value 0f 1.6 × 10^-3^ mm^2^/s is quite useful cut off point. (Accuracy 86%). ADC value and cut off point is good diagnostic tool but it has certain pitfalls. It is known that there is considerable overlap of values for benign and malignant lesions. Therefore, during interpretation of ADC values, conventional MRI sequences and clinical data should also be kept under focus^17^.

Another study by Yang DM et al.^18^ involving 97 patients, 137 FLLs were seen. Among these 97 patients, sixty patients were determined to be having 96 malignant FLLs. Histopathologic examination concluded diagnosis in these as HCCs in 52 patients, two cholangiocarcinomas, one metastatic lesion, and one hemangioendothelioma. Remaining thirty-seven out of 97 patients were found to be having 41 benign FLLs. On histopathological examination of these benign FLLs, “one adenoma, one angiomyolipoma, one ectopic adrenal adenoma, two inflammatory pseudotumor, one hepatic pseudolipoma , two FNHs(focal nodular hyperplasia) were diagnosed. The diagnosis of remaining benign solid FLLs was concluded clinically according to the standard of reference”.^18^

In a study by Suresh et al.,^19^ in India, it was stated that “MRI has 100% sensitivity and 93.55% specificity for malignant lesions, and for benign lesions it has 93.55% sensitivity and 100% specificity”. These slightly higher sensitivity and specificity percentages may be explained by the fact that in addition to DW \ADC mapping, contrast agent was also used in that study.

Hou ZB et al.^20^ worked on DW imaging in patients having hepatocellular carcinoma in background of liver cirrhosis. He also combined SWI (susceptibility weighted imaging) with DW Imaging. Results showed that “Coincidence rate was significantly higher (96%) as compared to conventional MRI (75%)” Federica et al.^21^ are of opinion that “employing MRI’s newer techniques having quantitative imaging features” “may be more helpful in providing a more useful classification of indeterminate liver lesions”

DW MRI scan has high sensitivity and specificity. Its accuracy was very high in predicting malignant lesion and differentiating it from benign lesion. By this modality more types of physical and chemical properties of normal, benign and malignant tissues can be recorded in a single MRI examination. Thus, DW MRI has capability of providing accurate and comprehensive diagnostic information. It also has additional advantage of having no ionizing radiation.

### Limitations

Main differentiating feature discussed in such studies is ADC values. Limitation include “considerable overlap of ADCs values seen between benign and malignant lesions. Depending only on ADC cut off values would lead to many false negative and false positive results, so final diagnosis needs to be concluded in combination with conventional MRI sequences and clinical data along with ADC values.^17^ Another limitation for the lesions in left lobe of liver is faced as motion artefacts caused by heart beating activity change the ADC values, thus measurements turn unreliable and add to limitations of study for lesions, as mentioned, detected in left lobe of liver.

## CONCLUSIONS

Diffusion Weighted MRI scan of liver with employment of its ADC values has high diagnostic accuracy and being highly accurate in making a diagnosis and differentiation of benign from malignant focal liver lesion would decrease need of invasive modality of histopathology.

### Authors contributions:

**J and ST:** Conceived and designed the study, included the patients. evaluated the HRCT scan findings, contributed to drafting and revising of article, final approval. She is also responsible for the integrity and accuracy of this study.

**SH:** Manuscript writing, critical revision, statistical calculations.

**H:** Data collection, processing data, data entry, drafting and revising the manuscript..
